# Posttraumatic Spinal Cord Injury without Radiographic Abnormality 

**DOI:** 10.1155/2018/7060654

**Published:** 2018-01-04

**Authors:** Kivanc Atesok, Nobuhiro Tanaka, Andrew O'Brien, Yohan Robinson, Dachling Pang, Donald Deinlein, Sakthivel Rajaram Manoharan, Jason Pittman, Steven Theiss

**Affiliations:** ^1^Department of Orthopaedic Surgery, University of Alabama at Birmingham, Birmingham, AL, USA; ^2^Department of Orthopaedic Surgery, Hiroshima University, Hiroshima, Japan; ^3^Royal Victoria Hospital and Royal Belfast Hospital for Sick Children, Belfast, UK; ^4^Department of Surgical Sciences, Uppsala University Hospital, Uppsala, Sweden; ^5^Department of Pediatric Neurosurgery, University of California, Davis, CA, USA; ^6^Regional Centre for Paediatric Neurosurgery, Kaiser Permanente Hospitals, Oakland, CA, USA; ^7^Great Ormond Street Hospital For Children, NHS Trust, London, UK

## Abstract

“Spinal Cord Injury without Radiographic Abnormality” (SCIWORA) is a term that denotes objective clinical signs of posttraumatic spinal cord injury without evidence of fracture or malalignment on plain radiographs and computed tomography (CT) of the spine. SCIWORA is most commonly seen in children with a predilection for the cervical spinal cord due to the increased mobility of the cervical spine, the inherent ligamentous laxity, and the large head-to-body ratio during childhood. However, SCIWORA can also be seen in adults and, in rare cases, the thoracolumbar spinal cord can be affected too. Magnetic resonance imaging (MRI) has become a valuable diagnostic tool in patients with SCIWORA because of its superior ability to identify soft tissue lesions such as cord edema, hematomas and transections, and discoligamentous injuries that may not be visualized in plain radiographs and CT. The mainstay of treatment in patients with SCIWORA is nonoperative management including steroid therapy, immobilization, and avoidance of activities that may increase the risk of exacerbation or recurrent injury. Although the role of operative treatment in SCIWORA can be controversial, surgical alternatives such as decompression and fusion should be considered in selected patients with clinical and MRI evidence of persistent spinal cord compression and instability.

## 1. Introduction

The acronym SCIWORA (Spinal Cord Injury without Radiographic Abnormality) was first defined in 1982 by Pang and Wilberger Jr. in a series of 24 children who suffered traumatic myelopathy with no radiographic evidence of fractures, dislocations, or malalignment of the spine [1]. Pang and Pollack described SCIWORA as a syndrome in which there are clinical signs of traumatic spinal cord injury (SCI) without overt traumatic vertebral column disruption as displayed by spine X-rays, computed tomographic (CT) scans, myelograms, and dynamic flexion/extension X-rays [2]. The first human magnetic resonance imaging (MRI) scan was done in 1977, and distinctive MRI signal patterns of acute SCI were first described in 1987 [3, 4]. Hence, the original report from Pang and Wilberger Jr. that introduced SCIWORA into medical literature did not include MRI in the definition of this syndrome; Pang acknowledged the diagnostic potential of MRI in patients with SCIWORA two decades later [5]. It might be sensible to ask, “Would SCIWORA still exist if MRI was readily available for use in acute SCI only a few years earlier?” The answer would probably be “Yes,” since MRI is never the first-line imaging modality in the setting of acute spinal trauma. Plain X-rays and CT are almost invariably performed before MRI, since MRI scans require more time, space, and patient transfer that might not be practical in emergency management of trauma patients.

SCIWORA is more commonly seen in the pediatric age group than in adults and involves the cervical spine more frequently than the thoracolumbar spine. The incidences have been reported between 13 to 19% and 10 to 12% of spinal injuries in children and adults, respectively [6–9]. SCIWORA is far more common in males than females [6, 8–10]. In a systematic review, Carroll et al. [10] documented that, of 368 pediatric patients with SCIWORA, approximately 68.5% were male, and 31.5% were female. Cervical spine was involved in 87% of the patients; thoracic spine was involved in 9.5%; lumbar spine was involved in 1.5%; and in 2%, the SCI spanned the cervical and thoracic levels. Evidence from the adult population indicating that thoracolumbar spine can also be involved with SCIWORA is limited to occasional case reports [11, 12]. The reasons for increased frequency of SCIWORA in the pediatric age group with a predilection for the cervical spinal cord include the large head-to-body ratio, increased mobility of the cervical spine, inherent ligamentous laxity, immaturity of neck musculature, incomplete ossification of the vertebrae, and shallow angulation of facet joints during childhood [2, 5, 12]. Interestingly, several studies showed that the upper cervical spine was more susceptible to SCIWORA in younger children than in older children, where the lower cervical spine is more commonly affected [2, 10, 13]. This finding is supported by the fact that the fulcrum of movement is at the upper levels of the cervical spine (between C2 and C4) in younger children and shifts to lower levels (C5-C6) in adolescents and adults [14, 15]. It is conceivable that SCIWORA is seen less frequently in adults as a result of age-related changes in bone morphology and a decrease in ligamentous laxity. Furthermore, the thoracic spine has a more stable and stiff structure compared to the cervical spine due to the surrounding rib cage and costovertebral articulations. Similarly, both the thoracic and lumbar spine have larger bony surfaces that increase axial loading capacity and stability [12].

## 2. Pathophysiology

Several mechanisms have been proposed to cause SCIWORA including spinal cord traction injury due to hyperflexion, extrinsic cord damage from hyperextension, and parenchymal cord damage resulting from edema or vascular injury [12]. The two-hit hypothesis is one of the possible pathophysiological explanations for delayed cord damage in patients with SCIWORA. After the primary injury from direct impact, a subsequent secondary insult to spinal cord parenchyma from complex cellular-level reactions to the primary injury can worsen the clinical picture [18]. Traumatic SCI may cause increased Na^+^ influx into the cells through voltage-gated channels, which may lead to increased H^+^ influx and intracellular acidosis through the activation of the Na^+^/H^+^ exchanger in an attempt by the cell to pump out accumulating intracellular Na^+^ [19]. Likewise, increased Na^+^ influx after SCI may cause reversal of Na^+^/Ca^++^ exchanger that results in an increase in Na^+^ extrusion and intracellular Ca^++^ accumulation with apoptosis of the neurons [18, 19]. These changes trigger intracellular events such as free radical-mediated cell damage, lipid peroxidation, and activation of membrane lipases. Consequently, a cascade of secondary inflammatory reactions, edema, and ischemia resulting in further spinal cord parenchymal insult can occur [18, 19].

## 3. Mechanism of Injury

The most common causes of injury in patients with SCIWORA are sports injuries, motor vehicle collisions, falls, and abuse [2, 10, 13]. In a series of 297 children who suffered from SCIWORA, Knox [13] demonstrated age-related variations in the mechanism of injury. Between 0 to 10 years, the most common cause of injury was found to be motor vehicle collisions (38–40%). However, sports injuries were the most common injury mechanism in children between 11 and 17 years of age (57%). In adult patients with SCIWORA, falls appear to be the most common mechanism of injury. Como et al. [20] reported that, of the 24 adult patients with SCIWORA, 67% had a mechanism of fall. In another study from Sharma et al. [9] of 12 adult SCIWORA patients, five (42%) were injured in motor vehicle accidents, and four (33%) fell from height. The difference between the two studies in terms of the most common mechanism of injury can be attributed to the difference in the number of patients included in each study (24 versus 12 patients, resp.). The literature includes sporadic publications reporting on thoracic SCIWORA cases in adults following motor vehicle accidents [11, 12]. Despite insufficient evidence, this may indicate the severity of the injury mechanism required to cause SCIWORA in the biomechanically more stable thoracic spine.

## 4. Diagnostic Evaluation

After the initial management in the field, diagnostic evaluation of patients with presumed SCI should start with a detailed history which can be possibly taken from eyewitnesses to determine the mechanism of injury [5]. More often than not, there are associated injuries of the head, thorax, abdomen, face, vasculature, pelvis, and the extremities. In a study of nationwide pediatric admissions, Knox [13] reported that 87% of patients with SCIWORA had associated injuries, and head trauma was the most common injury (between 28 to 64%), followed by orthopaedic injuries (10%), facial injuries (9%), thoracic injuries (9%), and gastrointestinal injuries (4%). It is imperative to detect and address these injuries, which can also provide clues to the mechanism of injury [5].

### 4.1. Clinical Findings

Clinical examination focusing on neurological findings may reveal a broad range of neurological deficits. Although clinical signs and symptoms can be observed from the moment of injury, neurological deficits may only become apparent several days after the injury due to second-hit phenomenon, edema, or a developing hematoma around the cord [21].

**Figure 1:**
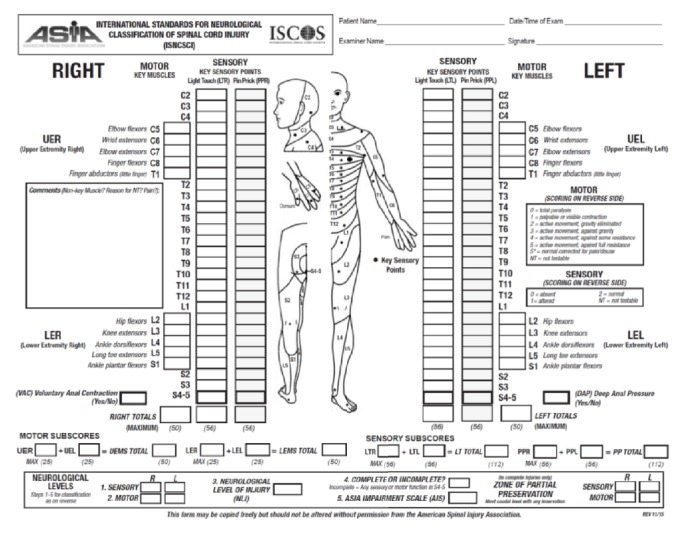
American Spinal Injury Association (ASIA) Injury Scale.* (Source: with permission from American Spinal Injury Association: International Standards for Neurological Classification of Spinal Cord Injury.)*

A thorough neurological examination immediately after the injury may indicate the level of SCI and help to monitor the progress of patients at later stages of their management. It is advisable to use one of the SCI scales, such as the American Spinal Cord Injury Association (ASIA) scale (Figure 1), and report the neurological examination findings as ASIA Impairment Scale (AIS) grades (Table 1) [22]. Published reports indicate that patients with SCIWORA may present with a wide range of neurological findings, including para/hemiparesis/plegia, paresthesia, changes in tendon reflexes, loss of bladder and bowel function, signs of anterior/central/posterior cord or Brown-Séquard syndrome in addition to local pain, sensitivity, abrasions, and bruising around the vertebral column [2, 11, 12, 17, 23].

In a retrospective case series from Martinez-Perez et al. [8] that included 48 adult patients diagnosed with SCIWORA, two patients had complete SCI with AIS grade A; five patients were documented as AIS grade B; 15 patients had AIS grade C; and 26 patients had AIS grade D SCI at admission. Neurological assessment of the patients at one-year follow-up revealed that neither of the two patients with complete SCI showed improvement after admission. All but eight patients (two grade B, two grade C, and 6 grade D) with incomplete SCI had improvement of at least one grade on AIS at one-year follow-up [8].

It should be kept in mind that neurological findings in SCIWORA patients may not always be prominent, and there may be fluctuations in severity.

### 4.2. Plain Radiographs and CT

**Figure 2:**
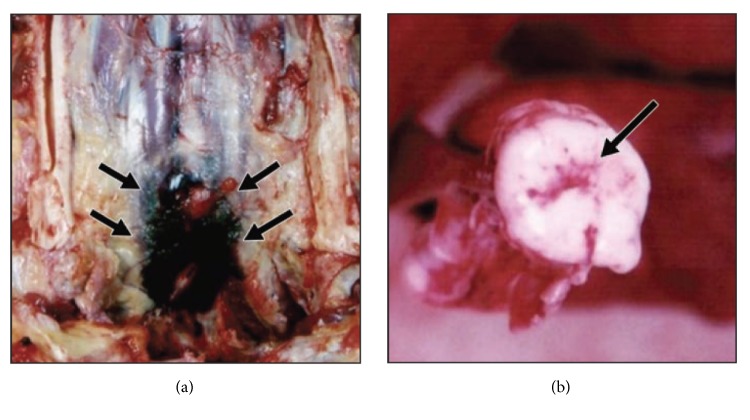
Autopsy photographs of a 44-year-old man found dead near his bicycle. Cause of dead was not explainable based on external examination and investigation. MDCT scan did not reveal any fractures, dislocations, or other signs of trauma. (a) Autopsy revealed perivertebral hemorrhage (arrows) anterior to C6 and C7. (b) Macroscopic axial autopsy photographs show hemorrhage (arrow) in cervical spinal cord at C5.* (Source: with permission from [16].)*

In all patients with traumatic SCI, anteroposterior (AP), lateral (LAT), and odontoid views of the cervical spine are obtained. Depending on the level of injury, AP and LAT views of the thoracic and lumbar spine are added. If plain films do not reveal any abnormalities, then thin-section CT scans with coronal and sagittal three-dimensional (3D) reconstructions are performed. By definition, neither plain X-rays nor CT will reveal any signs of vertebral column fractures, dislocations, or malalignment in patients with SCIWORA syndrome despite the neurological findings of traumatic SCI in clinical assessment. In a postmortem study with 30 cases whose autopsy findings indicated gross or microscopic injuries to the spinal column or cord, Makino et al. [16] found that six patients (SCIWORA group) had no postmortem multidetector CT (MDCT) scan findings suggestive of direct or indirect trauma to the cervical spine. Although 58% (14/24) of non-SCIWORA cases had MDCT-detectable perivertebral hemorrhage, none of the SCIWORA subjects had hemorrhages detectable in postmortem MDCT images (Figure 2). The results of this study point out that even using a more advanced CT imaging technique such as MDCT may not provide conclusive evidence of spinal cord injury in SCIWORA patients [16].

### 4.3. Dynamic Imaging

If standard AP and LAT plain X-ray and CT images do not reveal any fracture or dislocation, the stability of the spine can be assessed by dynamic flexion and extension radiographs. Although conceptually dynamic imaging might be seen as an alternative modality in the diagnostic algorithm of SCIWORA patients, current evidence does not provide enough support for its routine use. Pang and Pollack [2] obtained dynamic cervical films during the first week after injury in 55 children with SCIWORA and noted that, in most, severe paraspinous muscle spasm prevented adequate flexion. The authors repeated the dynamic films after spasm had subsided and showed late stability in only one patient who had anterior subluxation of C4 on C5 that was masked on previous studies due to spasm [2].

Several investigators have studied the role of dynamic imaging in spinal clearance of obtunded trauma patients and their findings revealed that flexion and extension views do not provide any advantage over CT and are not cost effective as a diagnostic modality in cervical spine clearance [24–26]. Considering that the patients in these studies were unconscious and in a relatively relaxed state [24–26], performing adequate dynamic imaging with meaningful results in conscious SCIWORA patients with paraspinous muscle spasm would be highly unlikely. Hence, we do not recommend using dynamic imaging in SCIWORA patients who already had negative plain X-rays and CT images.

### 4.4. MRI

The advent of MRI provided superior visualization of the soft tissue structures and enabled better recognition of the pathologies involving intervertebral disks, ligaments, and neural tissues including the spinal cord and nerve roots. As a result, MRI has become the gold standard diagnostic imaging modality in patients with presumed SCI [27]. There are characteristic pathomorphological soft tissue changes in SCIWORA patients that could only be detected using MRI but not in plain films or CT images including spinal cord hematomas, transections, discoligamentous injuries, spinal cord edema, and compression [28–31].

Machino et al. [32] studied MRI examinations from 100 SCIWORA patients. The authors detected changes in signal intensity that could be due to spinal cord hemorrhage, contusion, or edema in 92% of the patients. Furthermore, the authors measured the range of signal changes based on the height of the C3 vertebral body from the patients' own sagittal MRI images and found that larger signal changes were predictive of more severe symptoms and poorer outcomes. Boese and Lechler [33] suggested grouping SCIWORA patients based on MRI findings. Patients with no detectable abnormalities in MRI were defined as Type I, and all the patients with detectable MRI abnormalities were included in Type II. The Type II patients were further divided into three groups: extraneural, intraneural, and coexistence of both intra- and extraneural abnormalities. The latter authors identified 36 studies, including 605 adult SCIWORA patients with reported MRI findings [33]. In 43 patients (7.1%), no MRI abnormalities were detected (Type I), while 562 (92.9%) had abnormal MRI scan results (Type II). Of these, 71 patients (11.7%) had extraneural; 223 patients (36.9%) had intraneural; and 268 patients (44.3%) had combined extra- and intraneural MRI abnormalities. Intraneural abnormalities included edema, hemorrhage, contusion, and partial or complete transection. Extraneural findings were disc herniation, ligamentum flavum bulging, prevertebral soft tissue swelling, or ligamentous abnormalities [33]. There is evidence in the literature suggesting that SCIWORA patients with no detectable MRI abnormalities usually have a better prognosis and recovery rate than the patients with MRI abnormalities [34, 35].

In some cases, repeating the MRI scan may reveal abnormalities which were not evident in the initial examination, or changes in the extent of the previously identified MRI abnormalities [23, 28, 36]. Liu et al. [28] performed MRI in 59 SCIWORA patients with neurological deficiencies at the cervical or thoracic level. Two patients with neurologic deficits were classified as normal on initial MRI. These patients had repeat MRI scans 72 hours after the initial trauma, which revealed positive MRI abnormalities, and they underwent surgical interventions. Schellenberg et al. [23] reported on an 18-year-old male who was involved in a car accident as the seat-belted driver. Although the patient presented with paraplegia, his initial plain X-rays, CT, and MRI of the spine were normal. A repeat MRI scan five days after the collision revealed a new abnormal signal (10 mm in size) at the level of T3-T4, representing spinal cord edema. Ouchida et al. [36] performed MRI scan of 68 SCIWORA patients within 48 hours after the injury and repeated the MRIs two weeks after the injury to measure the changes in signal intensity and range. Repeat MRI scans revealed higher-grade signal intensity in 24 patients and attenuation in range of signal intensity as measured based on C3 vertebral height. Moreover, there was a significant negative correlation between the signal intensity grade and range and clinical symptom severity at two weeks. This negative correlation was absent in the acute MRI. The authors suggested that “delayed MRI can provide useful information about the state of the spinal cord after the acute phase…” [36].

**Figure 3:**
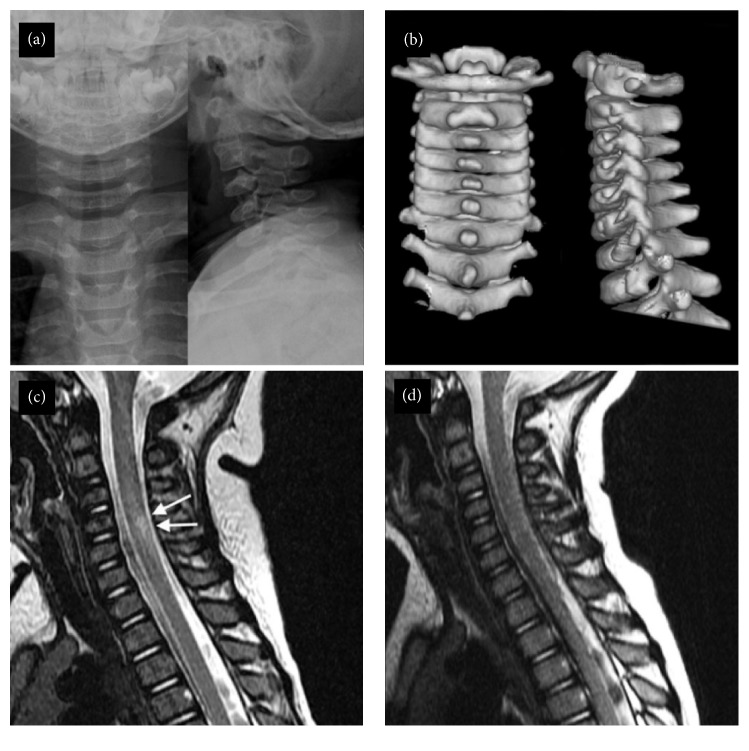
A 12-month-old female infant presented with nausea, vomiting, and drowsiness to emergency room after falling from a height of less than 30 cm. She had no neurological deficit at presentation, and cervical spine plain radiographs (a) and CT with 3D reconstruction (b) showed no abnormal findings. (c) Seven days after the injury the patient developed right sided hemiparesis and cervical MRI revealed increased intensity (arrows) in the T2-weighted images at the level of C6. (d) Repeat cervical MRI one month later shows that increased signal intensity has disappeared. The patient continued to improve neurologically until 24 months after the injury and returned to near-normal.* (Source: with permission from [17].)*

Regardless of the timing or number of scans, MRI appears as the sole diagnostic modality that may aid orthopaedic surgeons in understanding the mystery of SCIWORA syndrome, which does not reveal any findings in plain X-rays and CT (Figure 3).

### 4.5. Somato Sensory Evoked Potentials (SSEPs)

SSEPs are signals generated by the nervous system in response to electrical stimulation of a peripheral nerve. Since the SSEP signals are series of waves that reflect sequential activation of neural structures along the somatosensory pathways, monitoring these signals by electrodes positioned along these pathways can aid in detecting any dysfunction from the level of the peripheral nerve, through the spinal root, spinal cord, brain stem, and thalamocortical projections, up to the primary somatosensory cortex. Moreover, recording signal changes both at the craniovertebral junction and at the cortex can help distinguish between a spinal cord injury and thalamic or cortical dysfunction. Although SSEPs are routinely used for intraoperative neuromonitoring during surgery of the spine, literature support for their use in patients with SCIWORA is quite limited [5, 37, 38]. Moreover, evidence shows that SSEP changes are highly specific but not equally sensitive indicators of postoperative/postinjury neurological deficits [39]. Pang [5] used SSEPs as an adjunct in the initial evaluation of children with presumed SCIWORA. In the study of 95 children with SCIWORA, 50 had both MRI and SSEP data. SSEP recordings obtained within 24 hours after injury were found to be slightly more sensitive than MRI in patients with persistent (88% versus 64%, resp.) or transient (39% versus 27%, resp.) neurological deficits. The author suggested “Normal SSEPs should not be counted against an abnormal neurological examination in deciding whether myelopathy is present. Thus, SSEP recording should be regarded as a special rather than a routine test within the diagnostic algorithm of SCIWORA.”

## 5. Treatment

Although Advanced Trauma Life Support (ATLS) protocols and the initial steps of resuscitation after traumatic SCI are universally accepted, the method utilized to approach patients diagnosed with SCIWORA may vary between institutions; no definitive treatment protocol has been established yet.

### 5.1. Nonsurgical Treatment

Since overt signs of spinal trauma, such as fractures and dislocations, are absent in SCIWORA, nonsurgical strategies, including immobilization and corticosteroid therapy, are the mainstay of treatment. Immobilization immediately after the injury and at the early stages is performed using hard collars for cervical SCIWORA, or restriction of patients' movements with bedrest and log-rolling for thoracolumbar SCIWORA [5]. After the general condition of the patient has improved and other systemic injuries have been addressed, based on the level of SCI, a cervical or cervical-thoracic brace or thoracolumbar orthosis is applied, and the patient is allowed to get out of the bed and walk. Braces or orthosis are used for a minimum of three months until the reassessment of the neurological condition. At three-month follow-up, a decision as to whether the patient should have another MRI is made on an individual basis. Although the latter is the most widely accepted immobilization protocol, some authors suggest using halter traction with a minimal weight for the first three weeks and immobilization in an extended cervical collar until three months after the injury [35]. Interestingly, Bosch et al. [40] reported 21 patients with recurrent SCIWORA; 14 of them sustained their repeat episode while still wearing a rigid type of cervical brace. The remaining seven patients had their second injury either in a soft brace or beyond the time of immobilization. The authors suggested, “Bracing and immobilization do not prevent recurrent SCIWORA or improve outcomes in minor or severe SCIWORA once instability had been properly ruled out.” They also stated that “…bracing is not uniformly indicated.”

It is imperative to note that evidence to date does not include any randomized controlled trials to prove the superiority of one practice or suggestion over another. However, immobilization of the spine until the spine tenderness clears, the neurologic examination has normalized, and MRI is negative for instability is the universally accepted initial nonsurgical treatment approach [5, 10, 21, 35, 36, 40]. Regardless of the immobilization type, all SCIWORA patients are advised to refrain from any physical activities that may increase the risk of reinjury for approximately six months [21].

Posttraumatic spinal cord damage results from both primary (impact of trauma itself) and secondary mechanisms (subsequent cellular events and inflammatory response) that start at the moment of the injury and go on for days and even weeks [18]. The rationale for the use of steroid therapy in patients with SCIWORA is to prevent or minimize the secondary mechanisms that may cause damage to spinal cord after a traumatic injury. Although there is not enough evidence supporting routine use of high-dose intravenous (IV) methylprednisolone in SCIWORA patients, some studies suggest potential efficacy after SCI if it is started within the first eight hours of trauma with additional benefit by extending the maintenance dose from 24 to 48 hours [41]. Hence, IV methylprednisolone bolus of 30 mg/kg within eight hours of injury, followed by infusion at 5.4 mg/kg/hr for the next 48 hours can be beneficial in improving outcomes. In most SCIWORA cases, IV steroid therapy is started before an MRI scan can be completed and any detailed information with regard to pathological findings is available. Martinez-Perez et al. [8] reported that none of their 48 SCIWORA patients received corticosteroids during their hospitalization. In a prospective study including 45 consecutive SCIWORA patients, Mohanty et al. [35] routinely gave IV methylprednisolone to all the patients for 48 hours. Sharma et al. [9] administered methylprednisolone to seven of their 12 SCIWORA patients and stated “…the number of patients is too small to comment on the efficacy.” The effect of high-dose IV methylprednisolone therapy on clinical outcomes in SCIWORA patients requires further investigation.

### 5.2. Surgical Treatment

There is controversy in the literature regarding surgical treatment of patients diagnosed with SCIWORA. Although the majority of published reports suggest significant improvement in neurological status without operative treatment [17, 28, 31, 35], surgical intervention can become necessary in selected cases if there are clear signs of instability with ligamentous injury and/or cord compression which does not improve [8, 9, 36]. In a series that included 48 adult SCIWORA patients, Martinez-Perez et al. [8] treated 14 patients operatively. Of those 14 patients, six were treated by an anterior approach, and eight underwent decompressive laminoplasty or laminectomy. There was an improvement of at least one point on the ASIA Impairment Scale in 86% of the patients who received operative treatment compared with 76% of the patients who were treated conservatively. In a systematic review, Carroll et al. [10] identified 433 pediatric SCIWORA patients, and there were records of treatment in 183 cases. Of those, only six were treated operatively without notable details in terms of operative indications, technique, or outcomes.

**Figure 4:**
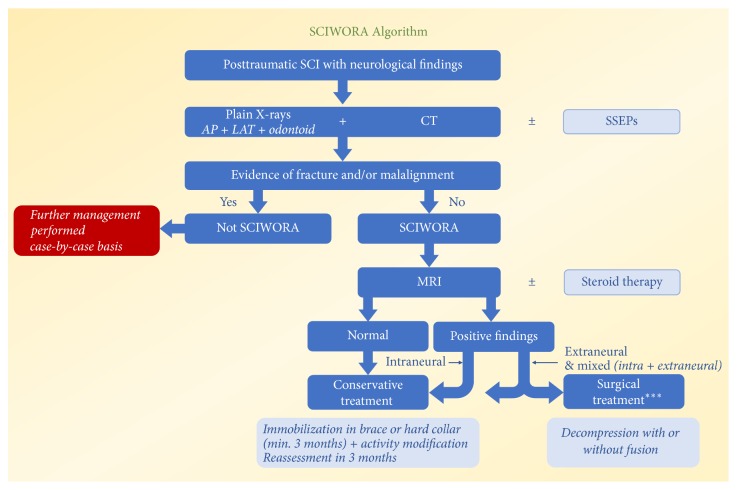
SCIWORA Algorithm. Pure intraneural MRI findings including edema or hemorrhage within the cord parenchyma is not an indication for surgery. Pure extraneural injury including severely injured ligaments or compression even without findings within the cord may be an indication for surgery. Patients with mixed extraintraneural MRI findings have the highest chance to require surgical treatment. Please note that there could be variations in diagnostic work up and treatment based on institutional or surgeons' preferences. ^*∗∗∗*^Main indications for surgical treatment are cord compression and ligamentous instability along with worsening or not-improving neurological findings.* (Courtesy of University of Alabama at Birmingham, Department of Orthopaedic Surgery, Birmingham, AL, USA.)*

Based on current evidence and our previous experience, surgical treatment is not recommended in SCIWORA patients with normal or pure intraneural MRI findings (i.e., cord edema or contusion without compression) regardless of patient's neurological status. Although clear MRI evidence of ligamentous injury, instability, spinal cord compression along with worsening, or not-improving neurological findings should be indications for surgical decompression with or without fusion, no controlled study to date has compared the outcomes of surgical treatment in SCIWORA patients with outcomes of nonsurgical treatment (Figure 4).

## 6. Prognosis

In general, most SCIWORA patients show remarkable improvement in neurological status after the injury, and surgical treatment is rarely justifiable. However, the main reason for the priority of conservative treatment in the management of SCIWORA patients is not the mild nature of the injury, but the absence of bony involvement and malalignment. Hence, the injury itself should be recognized as dreadful, and the prognosis can be dismal with devastating complications such as permanent neurological impairments and death [2, 16].

The two main predictors of prognosis after SCIWORA are the initial neurological status and MRI findings [2, 5, 32, 34]. Pang [5] suggests that neurological status at admission is the only predictor of long-term outcome in children with SCIWORA. Children with complete SCI rarely improve. Those with severe but incomplete SCI often improve but seldom regain normal function. In Pang's experience and based on several other reports, patients with mild to moderate initial neurological deficits may have more chance to attain full recovery [5, 8, 28].

Martinez-Perez et al. [8] reported that, at one-year postinjury follow-up, complete recovery in neurological status (AIS grade E) was achieved only in patients with incomplete neurological injury (AIS grades C and D) at admission. Although this study could not demonstrate a significant association between the neurologic impairment at admission with recovery, their results indicate a tendency for the less-severe SCIs to recover completely, while the patients with complete SCIs failed to show any progress. The lack of statistical significance can be explained by limited patient numbers and unequal distribution of the patients to groups with different AIS grades due to retrospective nature of the study.

Correlation between the MRI findings and prognosis has been the focus of several investigators [9, 28, 32, 34–36]. Mohanty et al. [35] showed significant negative correlations (*P* < 0.05) between the length of MRI changes in the spinal cord and the recovery rate (−0.026) as well as the final motor score (−0.042). There was a significant negative correlation between the length of prevertebral hyperintensity in MRI and AIS at the time of presentation (*P* < 0.001), final follow-up (*P* < 0.001), and the rate of recovery (*P* < 0.001). Authors reported that SCIWORA cases with normal MRI findings and spinal cord edema showed a higher mean recovery rate at two years after the injury (95.56 ± 12.54 and 87.70 ± 21.67, resp.). The rates of recovery in patients with MRI findings of cord contusion and cord swelling were the lowest among the groups (48.72 ± 42.08 and 39.42 ± 1.68, resp.), and the differences in recovery rates between different spinal cord changes were statistically significant (*P* < 0.05). Supporting these results, Boese et al. [34] showed that the mean improvement of AIS grade in SCIWORA patients with no MRI abnormalities was higher compared to those with detectable MRI abnormalities (1.5 versus 0.9, resp.). These authors also noted that all the patients who required surgical decompression were presented with simultaneous extra- and intraneural MRI findings.

Although long-term prognosis (over two years) of SCIWORA patients has not been studied extensively in large patient groups, expecting further improvement in neurological status after the first two years may not be realistic. However, worsening in the long-term due to recurrent injuries and/or development of deformities has been reported by several authors [40, 42]. Bosch et al. [40] had 21 patients with recurrent SCIWORA; of those, 20 were older than eight years of age at the time of initial injury. Furthermore, recurrences occurred up to three years after the initial event. The majority of recurrences happened during sports activities, and this may explain the higher recurrence rate among children over eight years of age. Yalcin et al. [42] reported four patients who developed progressive neuromuscular scoliosis due to SCIWORA. Spinal deformities were first noticed at a mean of 17 months after the initial injury that led to surgical interventions at a mean of 6.5 years.

## 7. Summary

SCIWORA is a syndrome that defines posttraumatic SCI in patients with neurological findings without any evidence of fractures or malalignment in plain X-rays and CT. This condition is more commonly seen in the pediatric age group, with a predilection for the cervical spine. Proposed mechanisms of injury include hyperflexion, hyperextension, and parenchymal cord damage resulting from edema or vascular injury that can occur as a result of sports injuries, falls, and motor vehicle collisions. Diagnostic evaluation starts with a detailed history and physical examination, followed by plain X-rays and CT. SSEPs can be done selectively; dynamic imaging does not provide any additional information and has been dropped from the diagnostic algorithm in SCIWORA. With its superior ability to reveal soft tissue pathologies and prognostic value, MRI is accepted as the imaging modality of choice in patients diagnosed with SCIWORA. Nonsurgical treatment with cervical brace or collar for a minimum of three months and restriction of high-risk activities for six months is the mainstay of treatment. Steroid therapy has not proven to be effective in SCIWORA patients. Based on evidence from studies in patients with SCI in general, it can improve the outcomes if started within eight hours of injury and continued for 48 hours. Surgical treatment should be reserved for patients with clear MRI evidence of extraneural findings including spinal cord compression, ligamentous injury, and instability, along with worsening or not-improving neurological findings. Prognosis of SCIWORA depends on the initial neurological deficit and extent of spinal cord injury as evidenced by MRI. Although neurological improvement in patients with complete neurological deficit at initial presentation is highly unlikely, most patients with incomplete neurological injury show improvement. Even so, permanent disabilities and deformities in the long-term are among the complications encountered by SCIWORA patients.

## Figures and Tables

**Table 1:** Summarized descriptions of ASIA Impairment Scale (AIS) Grades A, B, C, D, and E. Please note that only patients with an initial SCI and neurological findings receive an AIS grade. *(Source: with permission from American Spinal Injury Association: International Standards for Neurological Classification of Spinal Cord Injury.)*

American Spinal Injury Association (ASIA) Impairment Scale (AIS)		
Grade	Description	
A	Complete	No sensory or motor function is preserved in the sacral segments S4-5

B	Sensory incomplete	Sensory but not motor function is preserved below the neurological level and includes the sacral segments S4-5 AND no motor function is preserved more than three levels below the motor level on either side of the body

C	Motor incomplete	Motor function is preserved at the most caudal sacral segments for voluntary anal contraction OR the patient meets the criteria for sensory incomplete status

D	Motor incomplete	Motor incomplete status as defined above, with at least half (half or more) of key muscle functions below the single NLI having a muscle grade ≥ 3

E	Normal	If sensation and motor function are graded as normal in all segments and the patient had prior deficits, then the AIS grade is E
